# MDSC targeting with Gemtuzumab ozogamicin restores T cell immunity and immunotherapy against cancers

**DOI:** 10.1016/j.ebiom.2019.08.025

**Published:** 2019-08-25

**Authors:** Livingstone Fultang, Silvia Panetti, Margaret Ng, Paul Collins, Suzanne Graef, Nagy Rizkalla, Sarah Booth, Richard Lenton, Boris Noyvert, Claire Shannon-Lowe, Gary Middleton, Francis Mussai, Carmela De Santo

**Affiliations:** a Institute of Immunology and Immunotherapy, University of Birmingham, Birmingham, UK; b Department of Anatomic Pathology, The Chinese University of Hong Kong, Hong Kong; c CRUK Birmingham Centre and Centre for Computational Biology, Institute of Cancer and Genomic Sciences, University of Birmingham, Birmingham, UK

**Keywords:** MDSC, Gemtuzumab, CD33, Cancer, CAR-T

## Abstract

**Background:**

Targeting of MDSCs is a major clinical challenge in the era of immunotherapy. Antibodies which deplete MDSCs in murine models can reactivate T cell responses. In humans such approaches have not developed due to difficulties in identifying targets amenable to clinical translation.

**Methods:**

RNA-sequencing of M-MDSCs and G-MDSCs from cancer patients was undertaken. Flow cytometry and immunohistochemistry of blood and tumours determined MDSC CD33 expression. MDSCs were treated with Gemtuzumab ozogamicin and internalisation kinetics, and cell death mechanisms determined by flow cytometry, confocal microscopy and electron microscopy. Effects on T cell proliferation and CAR-T cell anti-tumour cytotoxicity were identified in the presence of Gemtuzumab ozogamicin.

**Findings:**

RNA-sequencing of human M-MDSCs and G-MDSCs identified transcriptomic differences, but that CD33 is a common surface marker. Flow cytometry indicated CD33 expression is higher on M-MDSCs, and CD33+ MDSCs are found in the blood and tumours regardless of cancer subtype. Treatment of human MDSCs leads to Gemtuzumab ozogamicin internalisation, increased p-ATM, and cell death; restoring T cell proliferation. Anti-GD2-/mesothelin-/EGFRvIII-CAR-T cell activity is enhanced in combination with the anti-MDSC effects of Gemtuzumab ozogamicin.

**Interpretation:**

The study identifies that M-MDSCs and G-MDSCs are transcriptomically different but CD33 is a therapeutic target on peripheral and infiltrating MDSCs across cancer subtypes. The immunotoxin Gemtuzumab ozogamicin can deplete MDSCs providing a translational approach to reactivate T cell and CAR-T cell responses against multiple cancers. In the rare conditions of HLH/MAS gemtuzumab ozogamicin provides a novel anti-myeloid strategy.

**Fund:**

This work was supported by Cancer Research UK, CCLG, Treating Children with Cancer, and the alumni and donors to the University of Birmingham.

**Research in Context**

**Evidence before this study**

Depletion of Myeloid-Derived Suppressor Cells (MDSCs), with unconjugated antibodies, in murine cancer models suggests that T cell responses can be reactivated against cancer. In humans, small molecule inhibitors which modulate MDSC intracellular suppressive mechanisms, have been trialled but have not shown the capacity to deplete MDSCs and restore T cell responses consistently across cancer groups.

**Added value of this study**

We identified that although human M-MDSCs and G-MDSCs are transcriptomically distinct, CD33 expression provides a surface target for both circulating and intra-tumoural MDSCs across cancer subtypes. Targeting of MDSCs with the anti-CD33 immunotoxin Gemtuzumab ozogamicin leads to immunotoxin internalisation, increased p-ATM, and MDSC cell death. The result is restoration of T cell proliferation and enhanced CAR-T cell proliferation and cytotoxicity against solid cancer targets.

**Implications of all the available evidence**

Our study suggests that Gemtuzumab ozogamicin provides the clinically relevant approach to deplete MDSCs in cancer patients, or pathological myeloid cells in HLH/MAS, and could overcome immunosuppressive microenvironments to reactivate T cell immunotherapy. These findings will be translated into a Phase II clinical trial (GOTHAM).

Alt-text: Unlabelled Box

## 1 Introduction

Cancer cells exist within an immune microenvironment, containing populations of cells which can coordinate and target the cancer or in contrast be subverted to promote cancer growth and survival [1]. Approaches to boost autologous T cell activity or engineer T cell immunotherapies have seen some dramatic clinical responses [2]. However in solid cancer and non-B-cell haematological malignancy trials clinical responses are disappointing, with poor Chimeric-Antigen Receptor T cell (CAR-T) expansion and persistence short-lived [[3], [4], [5]]. The failure is mainly due to an immunosuppressive microenvironment mainly created by Myeloid-Derived Suppressor Cells (MDSCs) in the tumours and blood of patients [6,7]. MDSCs may suppress T cell activity through diverse mechanisms including expression of immune checkpoint surface molecules, release of nitric oxide or reactive oxide species, production of immunomodulatory cytokines, or the consumption of amino acids [[6], [7], [8], [9]]. Myeloid cells also play critical roles in driving other diseases. Haemophagocytic Lymphohistiocytosis (HLH) and Macrophage Activation Syndrome (MAS) are a spectrum of rare conditions which may be familial or secondary to cancer, infection, or autoimmunity. They result in cytopenias, T cell activation, Natural Killer cell dysfunction and severe, life-threatening systemic inflammation marked by fever, high ferritin and hypertriglcyeridemia [10,11]. Expansions of myeloid cells which secrete pro-inflammatory factors such as IL-6, Il-18, and Il-1β is central to the underlying pathology, yet to date no therapies have directly targeted these cells contributing to a high mortality [12].

In murine models monocytic MDSCs can be readily defined by their expression of surface antigens such as Ly6C or CCR2 [13]. Administration of targeted antibodies that specifically deplete murine MDSCs, results in reactivation of anti-cancer T cell responses and tumour resolution – proof of principle that antibody targeting of these cells can have a dramatic and helpful effect on immunity [14]. Although markers for human MDSCs have been identified, such as CD10 or LOX1, expression is subtype-specific and clinical agents against these molecules are not well developed [15,16]. Furthermore clinical approaches to remove human MDSCs have been limited by complexities of MDSC characterisation, poor correlation between murine models and patients, and their ability to suppress T cells through multiple mechanisms [17]. One of the most clinically successful methods for selective cell depletion is the use of immunotoxins – antibody-toxin conjugates which induce cell-specific death [18]. Here we investigate potential clinically relevant targets for depleting MDSCs to reactivate T cell immunity.

## 2 Methods

### 2.1 Patient samples

Heparinized blood samples were obtained from adult patients with cancers of the lung (*n* = 21), pancreas (*n* = 7), colon (*n* = 36), brain (n = 7), head and neck (*n* = 8), prostate (*n* = 10), breast (*n* = 12), melanoma (*n* = 5) and paediatric patients with Wilms' (n = 5), neuroblastoma (*n* = 31), Ewing's (*n* = 2), non-Hodgkin's lymphoma (n = 2), rhabdomyosarcoma (n = 2) at diagnosis, prior to treatment. Blood from healthy donors (*n* = 41) was obtained at the University of Birmingham, UK. Healthy leukocyte cones were provided by the NHSBT Blood Bank (Birmingham, UK).

### 2.2 Study approvals

In accordance with the Declaration of Helsinki, patient samples were obtained after written, informed consent prior to inclusion in the study. Adult patient samples were collected through the University of Birmingham's Human Biomaterials Resource Centre (HBRC, Birmingham, UK). HBRC is licensed by the Human Tissue Authority to collect process and store project-independent human samples for biomedical research. Samples collected by HBRC are released under Research Tissue Bank ethical approval by the North West Research Ethics Committee, Haydock Park (Ref 15/NW/0079). Samples from Birmingham Children's Hospital were collected following Regional Ethics Committee (REC Number 10/H0501/39) approval.

### 2.3 Flow cytometric analysis of whole blood

All samples were processed within 12 h from collection. Whole blood was lysed prior to staining using the RBC Lysis solution (Qiagen, Germany) according to manufacturer's specification. Immune populations were identified by staining with fluorophore-conjugated anti-CD11b, anti-CD33, anti-CD3, anti-HLA-DR, anti-CD45, anti-CD68, anti-CD206, anti-CD163, (BioLegend), anti-CD14, anti-CD15 (eBioscience) antibodies on ice for 30 min. Fluorescence data was acquired using BD Accuri C6 (BD Biosciences), Cyan and/or CytoFLEX (Beckman Coulter) cytometers. Normalised population statistics –including the median fluorescence intensities (MFI) were determined using FlowJo (BD Biosciences, formerly developed by FlowJo LLC).

Where indicated cell death was assessed by propidium iodide staining of cells after 72 h incubation with Gemtuzumab ozogamicin (Gift from Pfizer).

### 2.4 Myeloid cell and lymphocyte isolation

Where indicated myeloid cells were isolated from peripheral blood using a Lymphoprep gradient (STEMCELL Technologies) and enriched from the white cell layer by positive magnetic assisted cell sorting (MACS) using anti-human CD14 Microbeads (Miltenyi Biotech) and MACS LS separation columns (Miltenyi Biotech). T-lymphocytes were obtained by negative selection after removal of myeloid cells. Cell purities of each distinct population of >98% was confirmed after isolation by flow cytometry using fluorophore-conjugated anti-CD14 or anti-CD3 antibodies.

### 2.5 RNA-sequencing

MDSCs were isolated from the peripheral blood of prostate, lung, head and neck, breast, and melanoma cancer patients at diagnosis (*n* = 3 per cancer) according to consensus guidelines using flow cytometry immunophenotyping and T cell proliferation assays as above [13]. RNA was derived from the MACS bead sorted CD14+ M-MDSCs and CD15+ G-MDSCs from cancer patients at diagnosis. Purity was checked by flow cytometry. Samples were prepared with the Illumina TruSeq RNA Sample Preparation Kit v2. They were sequenced on the Illumina HiSeq2000 platform using TruSeq v3 chemistry, over 76 cycles. Sequencing reads were aligned to GRCh37 human genome using STAR RNA-Seq aligner software [19]. Reads mapping to transcripts were counted by the same software. Normalisation of read counts and differential expression analysis comparing M-MDSCs and G-MDSCs was performed using DESeq2 R Bioconductor package [20].

### 2.6 Immunohistochemistry and scoring

A tissue micro-arrays (TMA) of 200 human tumours (*n* = 40 cases each of non-small cell lung carcinoma, prostate adenocarcinoma, breast invasive ductal carcinoma, colon adenocarcinoma, and pancreas duct adenocarcinoma) and normal control tissues (US Biomax) were deparaffinised and rehydrated following quality control to confirm diagnosis and antigen preservation. Tumours were stained on a Ventana Discovery Ultra automated system, according to manufacturer's protocol. Heat-induced antigen retrieval was performed with cell conditioning 1 buffer (CC1), pH 8.5 (Ventana). Protein blocking was applied with Background Sniper (Biocare Medical, Concord, CA). Staining with anti-human CD33 (Abcam) was performed at 37 °C, followed by the addition of secondary antibodies (Discovery anti-Rabbit HQ) using the Novolink Polymer Detection System (RE7280-K, Leica). Primary antibody incubation were carried out overnight at 4 °C and tissue sections were counterstained with haematoxylin and mounted in DPX (VWR). To assess nonspecific staining, samples were similarly treated but the primary antibodies omitted and replaced with isotype specific IgG (Vector Labs Peterborough UK).

Paraffin-embedded tissue sections of bone marrow trephines from 8 cancer-associated HLH patients at diagnosis were deparaffinised and rehydrated. Antigen retrieval was performed in 50 mM Tris/2 mM EDTA pH 9.0 using a Philips Whirlpool Sixth Sense microwave on a steaming program. Staining with anti-human CD33 (Abcam) was performed using the Novolink Polymer Detection System (RE7280-K, Leica). Primary antibody incubation was performed overnight in a cold room. Sections were counterstained with Gill Nr 3 haematoxylin (Sigma Aldrich) and mounted in Aquatex (Merck).

Antigen expression in immunohistochemistry sections were assigned independently by experienced pathologists. Briefly, to evaluate the immunostaining intensity in the tumour and bone marrow stroma each slide was examined on an Olympus BX51 microscope. Representative 400× magnification fields of at least 100 tumour cells were selected and photographed with an Olympus DP70 camera and accompanying image software. Fields were assigned an antigen staining intensity score of 0 = negative, + = weak, ++ = moderate, +++ = strong.

### 2.7 Gemtuzumab ozogamicin

MDSCs were seeded in complete medium at a density of 1 × 10^6^ cells/well of a 12 well plate. Unless otherwise stated, Gemtuzumab Ozogamicin (GO, Pfizer) was added at a concentration of 1 μg/ml and incubated for 48 h at 37 °C and 5% CO2. Analysis of cell death was monitored via propidium iodide (PI) (Sigma) uptake quantified on the CyTOFLEX flow cytometer. The cytotoxicity of unconjugated gemtuzumab antibody (Absolute Antibody) (2 μg/ml) and gemtuzumab ozogamicin (2 μg/ml) was similarly compared. For drug internalisation assays, GO was covalently labelled to AlexaFluor-647 fluorophore, with the Alexa Fluor Protein Labeling Kit (Life Technologies, Carlsbad, USA) as per the manufacturer's instructions. 1 μg/ml of labelled GO was added to cells and incubated on ice for 30 min to allow binding to the CD33 receptor, then at 37 °C at different time points for internalisation. Membrane-bound non-internalised drug was stripped using stripping buffer (0.2 M Glycine HCl, pH 2.2) and the cells analysed by flow cytometry. The MFIs of AlexaFluor-647 was determined using FlowJo (BD Biosciences, formerly developed by FlowJo LLC).

### 2.8 T lymphocyte proliferation assay

2 × 10 [5]/well of T cells were cultured in 96-well flat bottom plates coated with anti-CD3 (OKT3) antibody (3 μg/mL) and anti-CD28 antibody (2 μg/mL) (both eBioscience), in 200 μL R10% supplemented with 0.1% β-mercaptoethanol (Thermo Fisher Scientific). Cells were for 4 days incubated at 37 °C, 5% CO_2_ and their proliferation determined by ^3^H-thymidine (Perkin Elmer Life Sciences) incorporation assay using a TopCount NXT Scintillation Counter (Perkin Elmer). The suppressive ability of GO treated or untreated MDSCs was determined by direct co-culture with T cells. The data is expressed as a percentage of T cell proliferation driven by antibody stimulation in the presence of MDSCs relative to T cell proliferation in their absence (100%).

### 2.9 ELISA

The concentrations of cytokines within the patient's plasmas at diagnosis, were quantified using a competitive enzyme-linked immunoassay according to the manufacturers' instructions. The following molecules were tested: G-CSF, TGFβ, VEGF (all R&D Systems), IL-10, IL-4, IL-13, IL-6, IL-15, GM-CSF (all BioLegend).

### 2.10 Transmission electron microscopy

GO-treated, patient-derived MDSCs were pelleted at a density of 1.5 × 10^6^ cells/tube, fixed in 2.5% glutaraldehyde and stained with 1% osmium tetroxide. The samples were dehydrated using ethanol and fixed in a mixture of propylene oxide and resin at 60 °C for 16 h. Sections of 80 nm thick resin and sample were then taken and embedded onto copper slot grids for visualization under transmission electron microscopy (TEM).

### 2.11 Cell lines

Ewing sarcoma cell line (SKNMC), neuroblastoma cell lines (SKNAS, KELLY, IMR-32 and LAN-1) and mesothelin positive lung cancer cell line (H1299M) were routinely cultured in RPMI 1640 medium. Colorectal cell lines (Caco-2, SW480), caecal adenocarcinoma cell line (SNUC5), the pancreatic carcinoma cell line of ductal cell origin (PANC1) were cultured in DMEM (Sigma). All cell line media was supplemented with 10% *v*/v fetal bovine serum (FBS, Sigma), 100 U/mL penicillin and streptomycin (Gibco), 1 mmol/l- sodium pyruvate (Gibco), and 2 mmol/l l-glutamine (Gibco). Cell lines were originally obtained from ATCC and validated for authenticity by DNA short tandem repeats in line with American National Standards Institute ASN-0002-2011 (Northgene). Culture supernatants were harvested 72 h later from culture flask maintained in an incubator at 5% CO_2_ in air and at 37 °C. To generate tumour-polarised MDSCs, healthy CD14^+^ cells from leukocyte cones were cultured for 48 h in tumour-conditioned supernatant as previously described [7].

### 2.12 Immunofluorescence

MDSCs were seeded unto sterile No.1 (13 mm diameter) glass coverslip-inserts (VWR) at 1 × 10^4^ per well of a 24-well plate and maintained overnight at routine culture or experimental conditions. Cells were stained with anti-CD33 PE, washed in ice-cold PBS, fixed in 2% Paraformaldehyde for 20 min at RTP followed by permeabilisation in 0.1% Triton X for 10 min. After permeabilisation coverslips were blocked in a blocking buffer consisting of 5% heat inactivated goat serum (HiNGS) in PBS for 1 h at RTP. Coverslips were then incubated for 1 h with an eFluor 660 conjugated anti human Phospho-ATM (Ser1981) antibody (Clone 10H11.E12 3G5, eBiosciences) diluted 1:100 in 5% HiNGS/PBS. The coverslips were washed air dried, then mounted in SlowFade gold antifade mountant with DAPI (ThermoFisher Scientific). Cells were examined by fluorescence microscopy using a Zeiss LSM 780 fluorescence confocal microscope and images acquired using ZEN software suite (Carl Zeiss Microscopy).

### 2.13 CAR-T cell functional assays

T cells were engineered to express chimeric antigen receptors (CAR) for Mesothelin, GD2 and EFGRvIII based on established protocols [21,22]. T cells from leukocyte cones were transduced with anti-Mesothelin, anti-EGFRvIII and anti-GD2 CAR-containing retroviruses 48 h post-stimulation with anti-CD3/CD28. Successfully transduced T cells were FACS sorted exploiting the truncated-CD34 tag added to the construct and purity was checked by flow cytometry.

Tumour cell expression of target antigens was confirmed by flow cytometry using antibodies against mesothelin (Santa Cruz) and GD2 (Biolegend). For EGFRvIII, glioma cells were first dissociated from fresh human tumours within 24 h of surgery. RT-PCR was used to detect EGFRvIII expression. RNA was extracted using an RNeasy Mini kit (Qiagen). cDNA was prepared using SuperScriptTM III Reverse Transcriptase (Invitrogen) following the manufacturer's instructions. The PCRproducts were analysed by gel electrophoresis on a 2% agarose gel and were visualised by staining with ethidium bromide. The primer sequences are as follows: Primer 1 (Forward: 5′-ATGCGACCCTCCGGGACG-3′ Reverse: 5′-ATTCCGTTACACACTTTGCGGC -3′) and Primer 2 (Forward: 5′-GAGCTCTTCGGGGAGCAG-3′ Reverse: 5′-GTGATCTGTCACCACATAATTACCTTTTCT-3′) [23,24].

For the chromium assays, CAR T cells were incubated with antigen-positive tumour cells and antigen-negative controls, at 100:1, 33:1, 11:1, and 3:1 ratios in a 4-h ^51^Cr-release assay [22]. CAR-T cells were subsequently co-cultured with CFSE-labelled target-bearing cells (i.e. anti-Mesothelin with H1299M, anti-EGFRvIII with tumour cells isolated from glioma brain tumours, anti-GD2 with LAN-1), in presence of GO-treated or untreated MDSCs. Viability and number of tumour and CAR-T cells were investigated by flow cytometry using PI staining.

### 2.14 Statistical analysis

Parametric student *t*-tests were used to determine the statistical significance of the difference in paired observations between groups (GraphPad Prism, USA). All *p* values are two-tailed and p values <.05 were considered to represent statistically significant events.

## 3 Results

**Fig. 1:**
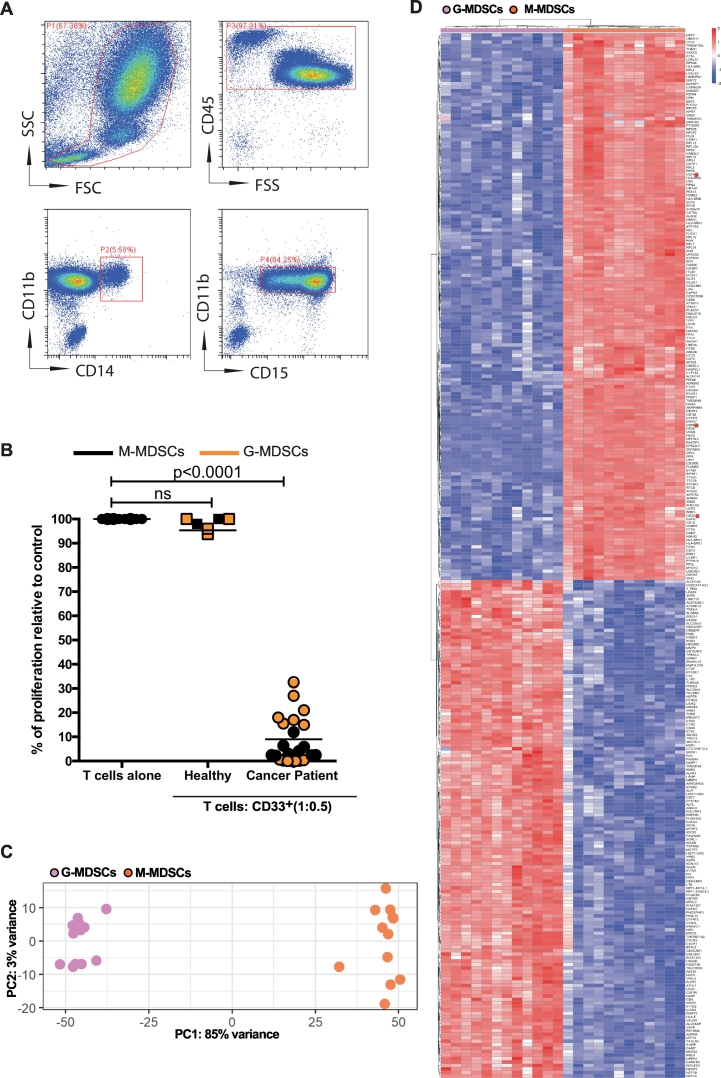
G-MDSCs and M-MDSCs from cancer patients have distinct transcriptomic profiles. A) Flow cytometry gating strategy, illustrating CD11b + CD14+ or CD11b + CD15+ myeloid cell populations in the blood of patients with cancer. Representative of *n* = 200 patient samples B) Sorted CD14+ and CD15+ myeloid cells from the blood of patients, but not healthy donors, suppress T cell proliferation consistent with M-MDSC and G-MDSC phenotype respectively. Co-culture ratio of 1:0.5 or T cells alone is shown. These cells were used for RNA-sequencing library generation. C) Principle Component Analysis for CD14+ M-MDSCs and CD15+ G-MDSC D) Heatmap of differential expression analysis comparing M-MDSC and G-MDSC samples from cancer patients. Top 300 genes shown.

To identify a MDSC surface target amenable for immunotoxin targeting CD14+ monocytic (M-MDSC) and CD15+ granulocytic (G-MDSC) MDSCs were isolated by immunophenotype and suppressive ability, according to consensus guidelines, from patients with different cancers (Fig. 1A and B) [13]. Generation of a RNA-sequencing library, to identify potential drug targets, revealed significant differences in the transcriptomic profiles of M-MDSCs and G-MDSCs (Fig. 1C and D). Analysis of the top 300 differentially expressed genes identified 3 potential targets for existing immunotoxins – CD74 [25], CD86 [26], and CD33 [27]. Of these, CD33 is the only one which clinically advanced in human trials. CD33 is a transmembrane Sialic-Acid-Binding-immunoglobulin-like lectin (SIGLEC) composed of a type 1 membrane protein with two immunoglobulin domains that binds sialic acid and intracellular immunoreceptor tyrosine-based inhibitory motifs (ITIMs) [28]. Knockout of the murine CD33 ortholog has no phenotype or role in defining murine MDSC populations [29]. Human CD33 on Acute Myeloid Leukaemia blasts has been successfully targeted by Gemtuzumab ozogamicin (GO), an anti-CD33 humanized antibody conjugated to calicheamicin in Phase III clinical trials [27]. We hypothesised that human MDSC CD33 could similarly be targeted, as a strategy across cancer subtypes.

**Fig. 2:**
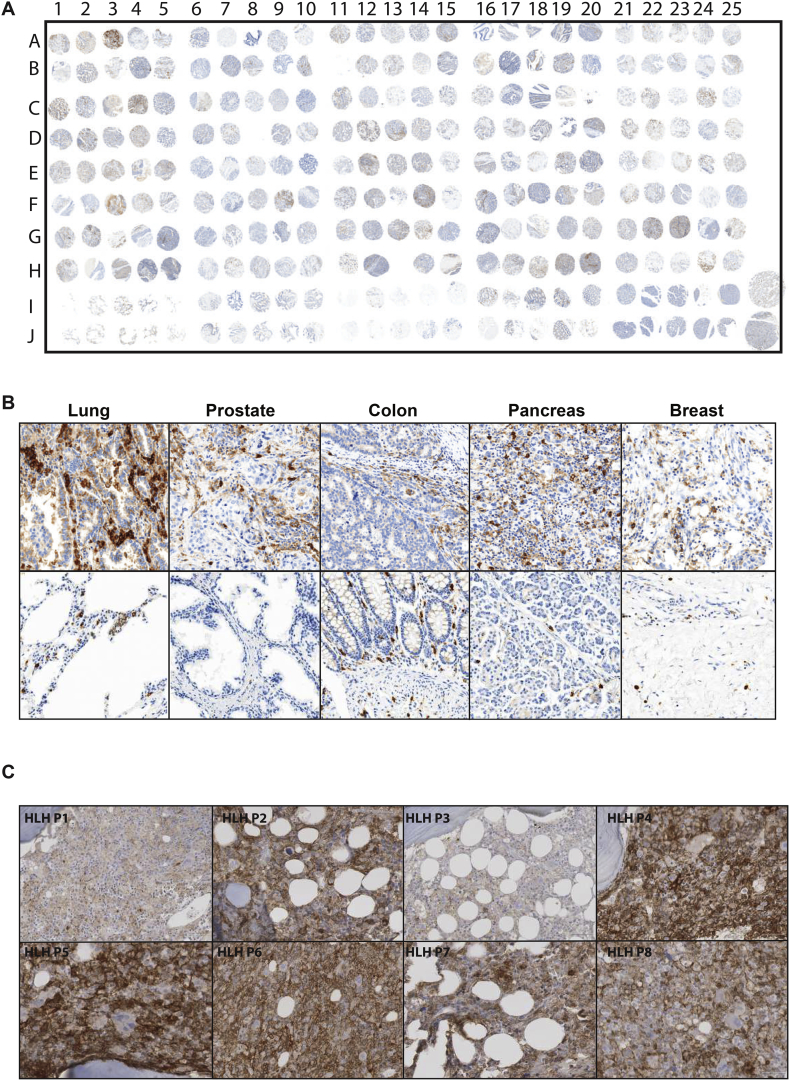
CD33+ MDSC infiltration in the tumours and bone marrow of cancer and HLH patients. A) Immunohistochemical analysis of tissue microarray (n = 200 cancer patients) B) Photomicrographs of representative CD33+ immunohistochemistry staining within lung, prostate, colon, pancreas, and breast tumours within the TMA (upper panels) and normal healthy control tissues (lower panels) C) Representative immunohistochemical staining of sections from bone marrows of HLH patients (*n* = 8) showing infiltration of CD33+ MDSCs.

**Fig. 3:**
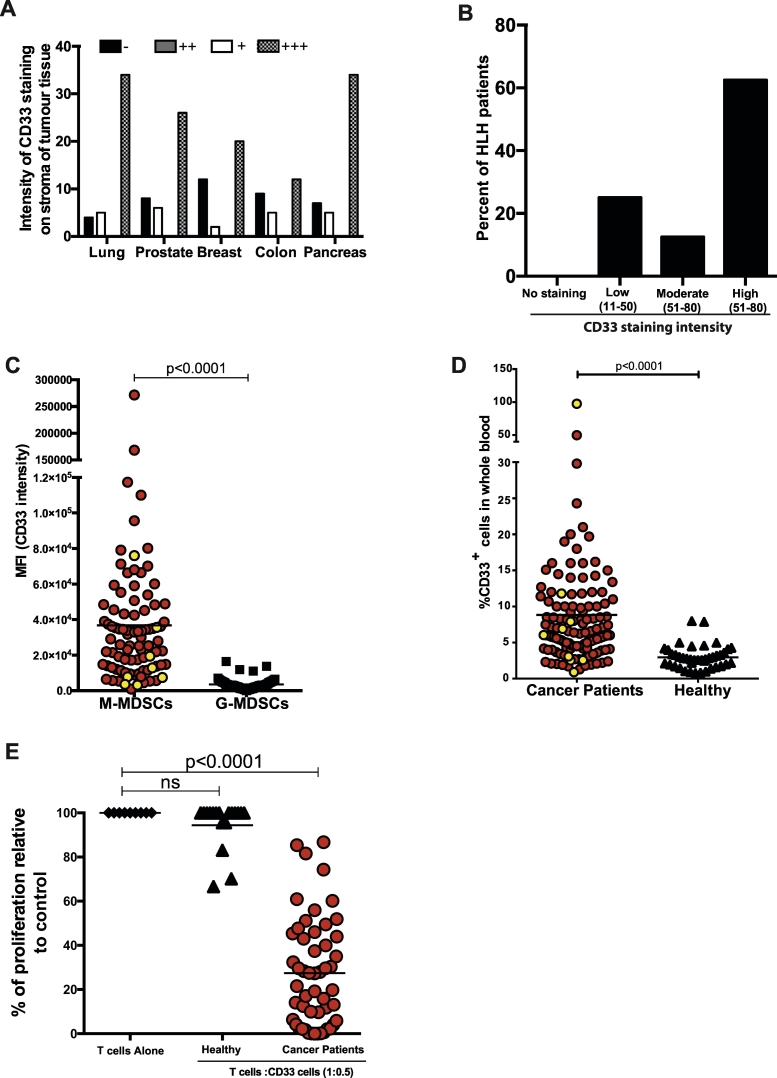
CD33 expression characterises the MDSC population in the tumours and blood of cancer patients. A) Intensity of CD33+ staining on MDSCs in the stroma of tumour subtypes (B) and bone marrow of HLH patients (C) Median Fluorescence Intensity of CD33 staining on M-MDSCs and G-MDSCs in the blood of cancer (RED) or HLH (YELLOW) patients (*n* = 81). D) Percentage of CD14 + CD33+ M-MDSCs in the blood of cancer patients (RED n = 81) and patients with secondary HLH (YELLOW,*n* = 7) E) T cell proliferation is suppressed following culture with CD33+ MDSCs from the blood of patients at diagnosis. (For interpretation of the references to colour in this figure legend, the reader is referred to the web version of this article.)

Examination of 200 patient samples revealed significant infiltrations of CD33+ myeloid cells in the tumour stroma compared to healthy tissues (Fig. 2A,B and Supp 1A,B). More rarely abnormal expansion and activation of myeloid cells can lead to a severe and life-threatening systemic inflammation - Haemophagocytic Lympho-Histiocytosis (HLH) or a Macrophage Activation Syndrome (MAS). In these rare patients we also identified a high frequency of CD33+ cells in bone marrow staining (Fig. 2C, Supp Fig. 2). The majority of cancer or HLH samples had high intensity of CD33 positivity (Fig. 3A and B). In the blood, CD33 intensity was greater on the M-MDSCs compared G-MDSCs (Fig. 3C) and this population is expanded compared to healthy controls (Fig. 3D). Culture of sorted CD33+ MDSCs confirmed their ability to suppress T cell proliferation (Fig. 3E), consistent with a reduction in peripheral T cells observed in patients at diagnosis (Supp Fig. 3A). Notably CD33+ cells sorted from the blood of healthy donors were not immunosuppressive. Thus CD33 is expressed on the MDSCs pathologically expanded in the blood and tumour tissues of adults and children with cancer and which create an immunosuppressive microenvironment.

**Fig. 4:**
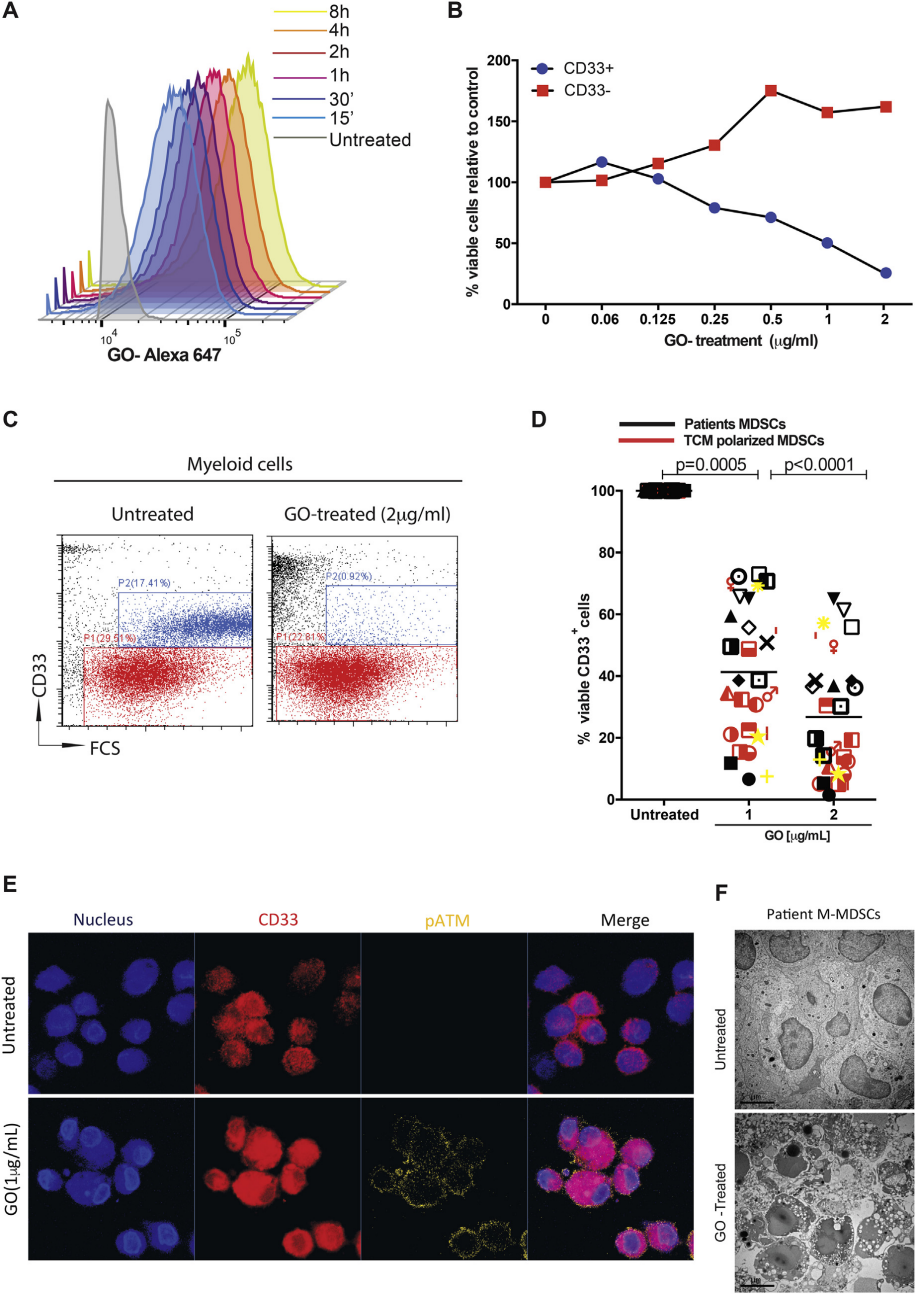
Gemtuzumab ozogamicin is cytotoxic to MDSCs. A) ALEXA-647-Gemtuzumab ozogamicin is rapidly internalised into MDSCs over time. Flow cytometric representation of 3 independent experiments. Gemtuzumab ozogamicin specifically depletes CD33+ MDSCs from the whole blood of patients ex vivo (BLUE), with no effect on the CD33- populations of cells (RED). Representative dose response curve of cell viability (B) and flow cytometry gating (C) shown D) Gemtuzumab ozogamicin (1 or 2 μg/ml) significantly reduces the viability of CD33+ patient-derived or tumour-polarised MDSCs from different cancer subtypes, as assessed by flow cytometry E) Confocal microscopy of GO-treated (1 μg/ml) CD33+ MDSCs from the blood of patients showing increased p-ATM F) Transmission electron microscopy shows loss of cell membrane integrity, nuclear condensation, and blebbing consistent with apoptotic cell death after 1 μg/ml GO treatment. (For interpretation of the references to colour in this figure legend, the reader is referred to the web version of this article.)

Incubation of CD33+ MDSCs from cancer patients with ALEXA647 labelled-GO confirmed binding predominantly to the M-MDSC population (Supp Fig. 3B), and rapid immunotoxin internalisation (Fig. 4A and Supp Fig. 3C). Although the unconjugated gemtuzumab antibody had minimal effect on survival (Supp Fig. 3D), Gemtuzumab ozogamcicin induced a dose-dependent decrease in viability (Fig. 4B, C, Sup Fig. 3D and 4A) of M-MDSCs from patients' PBMCs or tumour-polarised CD33+ myeloid cells (Fig. 4D), with no effect on CD33- cells. Suppressive tumour polarised CD33+ cells (Supp Fig. 4C) down-regulated HLA-DR and upregulated CD68 consistent with a M1-like phenotype (Supp Fig. 4D). GO treatment leads to increased pATM (Fig. 4E, Supp Fig. 5A) consistent with calicheamicin induced DNA-damage [30]. Electron microscopy revealed a loss of cell membrane integrity, nuclear condensation, and blebbing marking apoptotic cell death (Fig. 4F and Supp Fig. 5B).

**Fig. 5:**
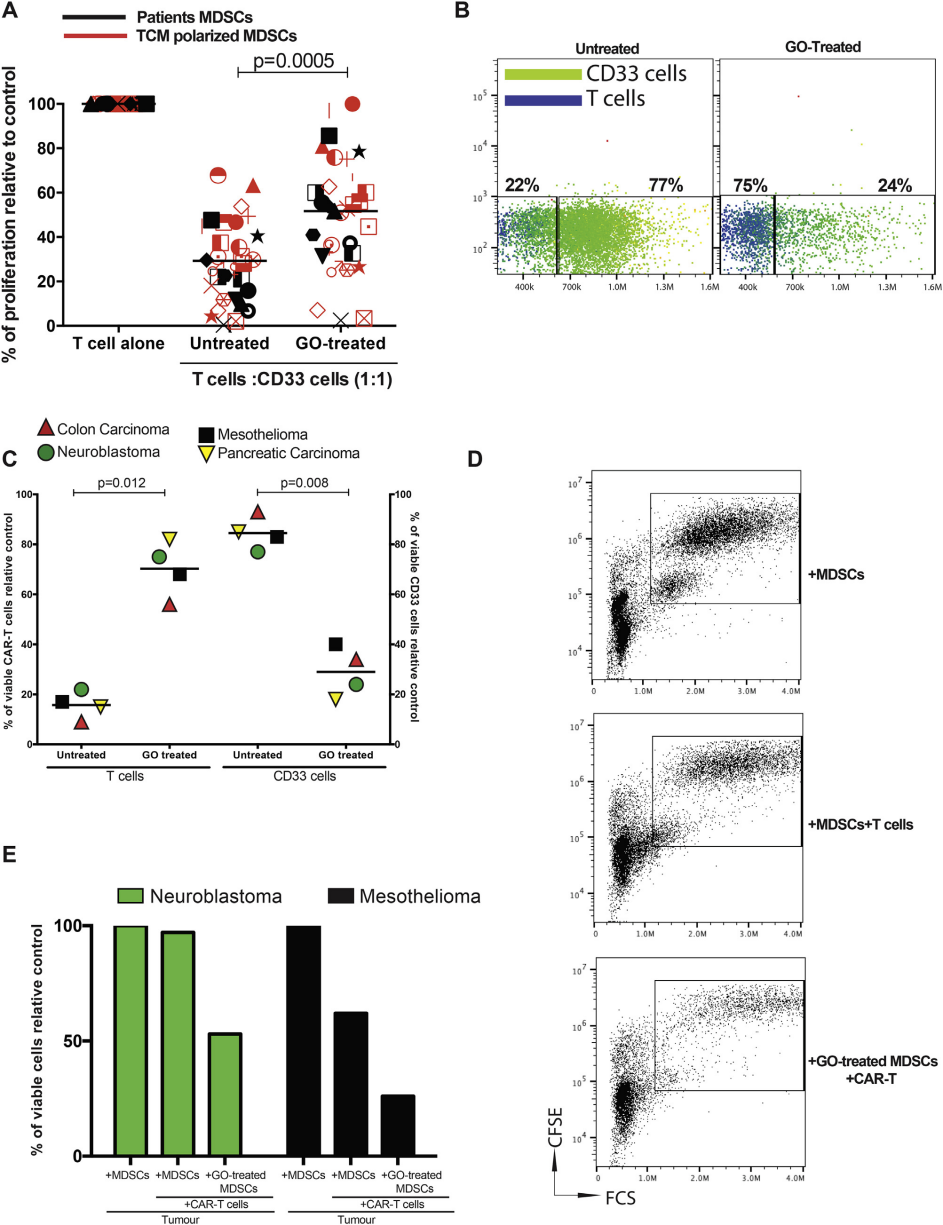
Gemtuzumab ozogamicin restores T cell and CAR-T cell proliferation and cytotoxicity. A) Treatment of patient-derived or tumour-polarised MDSCs from different cancer subtypes with Gemtuzumab ozogamicin restores T cell proliferation in co-culture as assessed by ^3^H-thymidine incorporation B) Representative flow cytometry gating showing the enhanced proliferation of CAR-T cells (BLUE) following the depletion of MDSCs by Gemtuzumab ozogamicin in tumour co-cultures C) Gemtuzumab ozogamicin depletion of MDSCs enhances CAR-T cell proliferation, as assessed by flow cytometry. Anti-mesothelin CART cells, anti-GD2 CART cells, and anti-EGFRvIII CART cells D)Representative flow cytometry gating on CFSE-labelled H1299M tumour cells in the presence of anti-mesothelin CAR-T cells and CD33 + MDSCs. E) Gemtuzumab ozogamicin depletion of MDSCs enhances CAR-T cell killing of target tumour cells, as assessed by flow cytometry. Representative of 4 independent experiments. (For interpretation of the references to colour in this figure legend, the reader is referred to the web version of this article.)

**Fig. 6:**
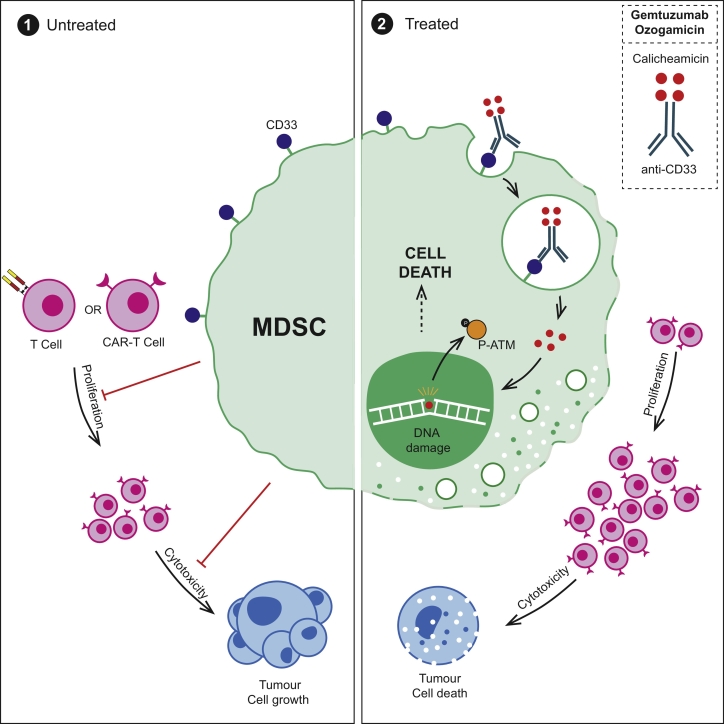
Schematic illustrating the capacity of MDSCs to suppress T cell and CAR-T cell proliferation and cytotoxicity. Treatment with Gemtuzumab Ozogamicin restores T cell and CAR-T cell proliferation, leading to enhanced tumour cell death.

Treatment of circulating or tumour-polarised MDSCs, with GO, restores T cell proliferation (Fig. 5A). The finding has potential clinical importance for CAR-T therapies against solid tumours, where CAR-T cell numbers in the blood and tumours fall rapidly post-infusion, despite the presence of target antigens. We hypothesised that circulating immunosuppressive cytokines from tumours could limit CAR-T expansion and anti-tumour activity. However with the exception of TGF-β [31], we found no consistent significant increases in IL-10, IL-4, Il-13, Il-6, GM-CSF, G-CSF, or VEGF in the blood that could account for the failure across cancer patients (Supp Fig. 6A-F). Instead systemic and intra-tumoural MDSCs may play a more prevalent pan-tumour inhibitory role [32]. CAR-T cells against 3 of the most common antigen targets were generated (Supp Fig. 6G) –Mesothelin, GD2 and EGFRvIII (Supp Fig. 7A, B,C). Antigen-specificity of CAR-T cell killing against corresponding tumour cell targets was first confirmed (Supp Fig. 7D,E,F). Mimicking patients' findings, MDSCs suppressed CAR-T cell proliferation, irrespective of the scFv, but this was overcome by GO-treatment(Fig. 5B,C). MDSCs also impaired CAR-T cell cytotoxicity. However GO-killing of MDSCs improved the effectiveness of each CAR-T construct, leading to a further significant reduction in viable mesothelioma and, neuroblastoma cells (Fig. 5D,E). Thus GO can kill MDSCs, overcome the immunosuppressive microenvironment, and provide a therapeutic boost to CAR-T cell activity (Fig. 6).

## 4 Discussion

The resurgence in T cell immunotherapy approaches for adult and paediatric cancers has highlighted the need for clinically relevant strategies against the underlying immunosuppressive microenvironment. One of the major mechanisms of tumour-immune escape, is through the expansion of immunosuppressive MDSCs [33]. It is well established that these cells may be significantly increased in the blood and tumours of adults and children with solid malignancies regardless of tumour type, and are associated with higher stage, metastatic disease, and a worse prognosis. These cells have been polarised by the tumour microenvironment to switch off autologous anti-cancer T cells responses and can impair both the manufacturing and efficacy of CAR-T cells [21,22,32].

One of the principle challenges in targeting human MDSCs remains their heterogeneous nature, with differences in immunophenotype and intracellular mechanisms of suppression both within the same patient (blood vs tumoural) and across different types of cancer diagnoses. Murine MDSCs can be readily characterised by immunophenotype, which has allowed for a detailed study of suppressive pathways and indeed transcriptomic changes [34,35]. However human MDSCs require a multi-step approach with relatively crude techniques including density centrifugation. To help identify new targets for clinical translation we generated a RNA-sequencing library from both M-MDSCs and G-MDSCs, to our knowledge the first such attempt at this strategy. It reveals clear separation of the MDSC at the transcriptomic level, confirming that these populations are indeed distinct. Although we focused on screening the library for potential clinical targets to deplete MDSCs, we suggest such data will also allow for an improved understanding of the underlying biology of M-MDSCs and G-MDSCs and could identify other strategies to isolate or modulate these cells.

Here we have identified how CD33 marks the MDSCs which are found in the blood and tissues of patients across cancers. Binding of sialic acid ligands to CD33 can induce a number of physiological function resulting in inhibition of cellular proliferation and activation, apoptosis, or modulation of cytokine release [36]. However Siglecs, such as CD22, CD33, Siglec-8, also provide an attractive target for antibody-based therapeutic due to their cell-specific surface expression and rapid internalisation kinetics [[37], [38], [39]]. CD33 may be targeted through unconjugated antibodies, however these antibodies are not usually cytotoxic and do not lead to sustained depletions of the target cells - minimising any therapeutic benefit [40,41]. Instead immunotoxins, such as Gemtuzumab ozogamicin, can induce a cell-specific cytotoxicity alone, without reliance on Antibody-Dependent Cellular Cytotoxicity. We show that the end result is a depletion, rather than a modulation, of the MDSCs, hence removing the immunosuppression regardless of the mechanism. Although CD33 may also be expressed on normal monocytes and macrophages in healthy individuals, critically it is the MDSCs which represent the bulk of myeloid-cells in the cancer patients who would receive Gemtuzumab ozogamicin. Furthermore we show that GO does not bind the CD33- populations of cells, thus immunity provided by granulocytes or T cells remains unopposed.

Myeloid cells also play critical roles in driving the rare but often fatal conditions of Haemophagocytic Lymphohistiocytosis and Macrophage Activation Syndrome [10,11]. Recently the diagnosis of HLH/MAS has become notable due to the use of immunotherapy approaches in cancer patients treated with Chimeric-Antigen Receptor T cells or antibodies [42,43]. Although it is recognised that myeloid cells secrete IL-1β or IL-6, modulate T and NK cells, or directly consume erythrocytes are central to the underlying pathology, no therapies have directly manipulated these cells [12]. Instead therapies have centred on multi-drug chemotherapies, T cell depletion, or inhibition of Il-1β or IL-6 [44]. As a rare and non-malignant disease few treatments have been developed which rationally target the underlying biological mechanisms, contributing to the poor prognosis for these patients (Supp Fig. 8).

Gemtuzumab ozogamicin has already completed Phase III adult trials and is subject to ongoing paediatric trials in children, with a manageable toxicity profile to date [45]. We suggest our findings have major clinical implications for the use of Gemtuzumab ozogamicin as an adjunct to chemotherapies and immunotherapies in adult and paediatric solid cancers. It is possible that patients with the highest frequencies of M-MDSCs, such as those with advanced or relapsed disease, may gain the most useful clinical effect from GO therapy by turning immunologically ‘cold’ tumours, into immunologically hot’ ones. Indeed in the context of CAR-T cells, co-treatment of Gemtuzumab ozogamicin could enhance CAR-T cell persistence and anti-tumour activity which has limited trial outcomes in solid tumours to date. To this end we will rapidly translate our preclinical findings to investigate the activity of Gemtuzumab Ozogamicin in HAemophagcytic lymphohistiocytosis (HLH) or Macrophage activation syndrome or relapsed/refractory solid tumours in an upcoming Phase II clinical trial (GOTHAM).

The following are the supplementary data related to this article.

Supplementary Fig. 1 CD33 + MDSCs in the tumours of cancer patients.
A) Increased frequency of CD33+ MDSCs in the tumour stroma of cancer patients, compared to healthy tissues, as assessed by immunohistochemical analysis of tissue microarray (*n* = 200 patients) B) Tissue Micro Array sample key. Supplementary Fig. 1

Supplementary Fig. 2 Bone marrow histology of patients with secondary Haemophagocytic Lymphohistiocytosis.
Immunohistochemical staining with Haematoxylin and Eosin in the bone marrow of patients (*n* = 8) with secondary Haemophagocytic Lymphohistiocytosis. Supplementary Fig. 2

Supplementary Fig. 3 Myeloid and T cell populations in the blood of patients.
A) Percentage of CD3+ T cells in the blood of cancer patients at diagnosis (*n* = 51) assessed by flow cytometry B) Flow cytometry gating of patient's whole blood demonstrating GO-ALEXA-647 staining of MDSCs. Representative of 5 individual experiments C) Gemtuzumab ozogamicin labelled with ALEXA-647 is internalised into MDSCs. Flow cytometric representation of internalised fluorescence over time. Representative of 3 independent experiments. D) The cytotoxicity of unconjugated gemtuzumab antibody (2 μg/ml) against CD33+ patient-derived MDSCs from different cancer subtypes, compared to gemtuzumab ozogamicin (2 μg/ml) and untreated controls, as assessed by flow cytometry with propidium iodide staining. Supplementary Fig. 3

Supplementary Fig. 4 Gemtuzumab ozogamicin has activity against MDSCs.
A) Dose-responses curves of Gemtuzumab ozogamicin cytotoxicity against CD33+ patient-derived MDSCs from different cancer subtypes, compared to untreated, as assessed by flow cytometry with propidium iodide staining B) Immunophenotyping of cancer patient and HLH patient blood demonstrating expansions of CD33 + CD14 M-MDSCs, by flow cytometry. Representative of *n* = 124 patients and 41 healthy donors C) T cell proliferation is suppressed following culture with CD33 + CD14+ tumour-polarised MDSCs. T cells and CD33 + CD14+ tumour-polarised MDSCs were co-cultured at a ratio of 1:0.5 and compared to CD33 + CD14+ monocytes. Mean of T cells and unpolarised MDSC proliferation shown as controls. D) The percentage of CD68+ CD14+ cells is increased following tumour polarisation, compared to the mean of unpolarised cells (RED). HLA-DR is also downregulated compared to unpolarised cells, as assessed by flow cytometry. Supplementary Fig. 4

Supplementary Fig. 5 Gemtuzumab ozogamicin leads to MDSC cell death.
A) Immunofluorescence staining of GO-treated (1 μg/ml) CD33+ MDSCs from the blood of patients showing increased p-ATM B) Transmission electron microscopy shows loss of cell membrane integrity, nuclear condensation, and blebbing consistent with apoptotic cell death after 1 μg/ml GO treatment. Supplementary Fig. 5

Supplementary Fig. 6 Systemic immunosuppressive cytokine environment in cancer patients.
A-F) ELISAs detecting cytokine concentrations in the blood of cancer patients at diagnosis (*n* = 50), compared to healthy controls G) Flow cytometry demonstrating efficiency of CAR-T transduction of T cells prior to enrichment, gating on tCD34. Supplementary Fig. 6

Supplementary Fig. 7 CAR-T cell cytotoxicity against antigen expressing tumour cell targets.
A) Expression of mesothelin by Meso-ACC cell line, as determined by flow cytometry B) Expression of GD2 by LAN-1 cell line, as determined by flow cytometry C) Expression of EGFRvIII by glioma tumour cells from a patient, as determined by RT-PCR. Chromium (^51^Cr) release assay demonstrating antigen- specific killing of tumour cell targets (RED) by CAR-T cells. Minimal killing is seen against tumour cells which don't express the corresponding antigen (BLUE). H1299M (Mesothelin positive), LAN-1 (GD2 positive, mesothelin and EGFRvIII negative), K562 (GD2 negative), Glioma (EGFRvIII positive). Supplementary Fig. 7

Supplementary Fig. 8 Schematic illustrating the potential of Gemtuzumab ozogamicin to target myeloid cells in HLH/MAS which release pro-inflammatory cytokines and drive pathological T cell responses. Supplementary Fig. 8

## Author contributions

F.M. and C.D.S. designed the study, supervised research, analysed data, secured funding and wrote the manuscript. F.M. additionally secured ethical approval and was chief investigator of the study. L.F. designed and performed research, S.P. performed research, S.B. performed research, R.L. performed research, P.C. performed research, M.N. performed patient samples, immunohistochemistry analysis and scoring, C·S-L, S.G., N.R. and G.M. provided patient samples. B.N. performed RNA-sequencing data analysis.

## Declaration of Competing Interest

The authors declare no conflicts of interest.
