# Supplementary figures and images for: MDSC targeting with Gemtuzumab ozogamicin restores T cell immunity and immunotherapy against cancers
Source: eBioMedicine. 2019 Aug 25;47:235–46. doi: 10.1016/j.ebiom.2019.08.025 (PMC6796554; doi:10.1016/j.ebiom.2019.08.025)

A

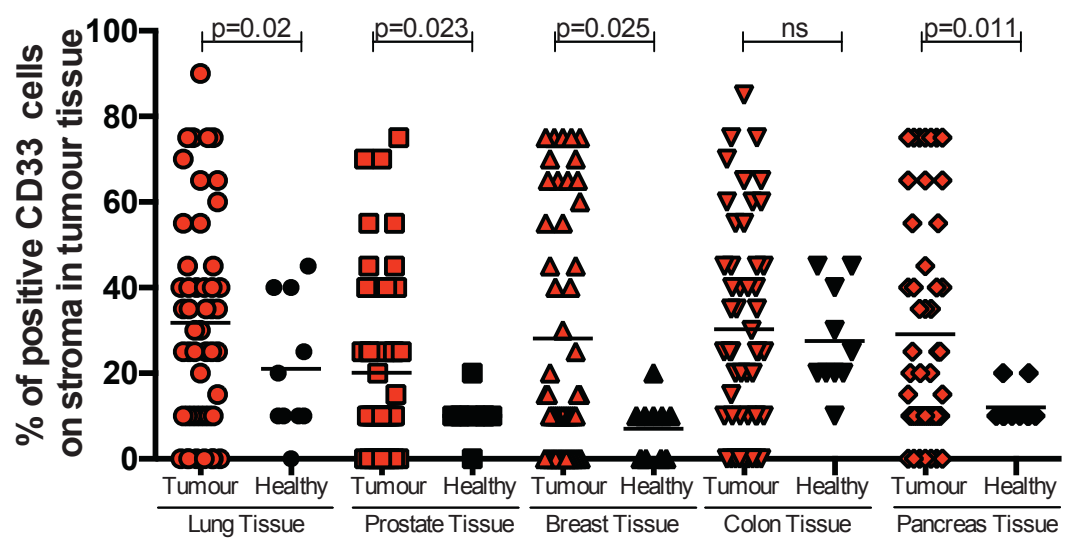

B

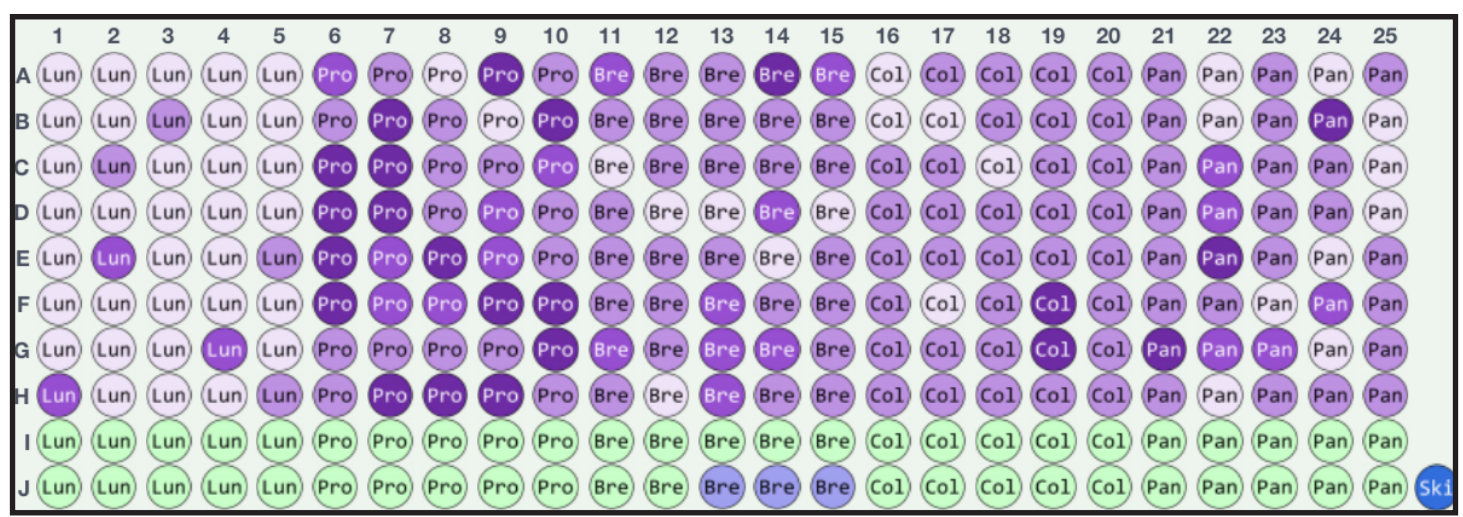

Supplement: Supplementary Fig. 1 — CD33 + MDSCs in the tumours of cancer patients. A) Increased frequency of CD33+ MDSCs in the tumour stroma of cancer patients, compared to healthy tissues, as assessed by immunohistochemical analysis of tissue microarray (n = 200 patients) B) Tissue Micro Array sample key. [file mmc1.pdf]

**A**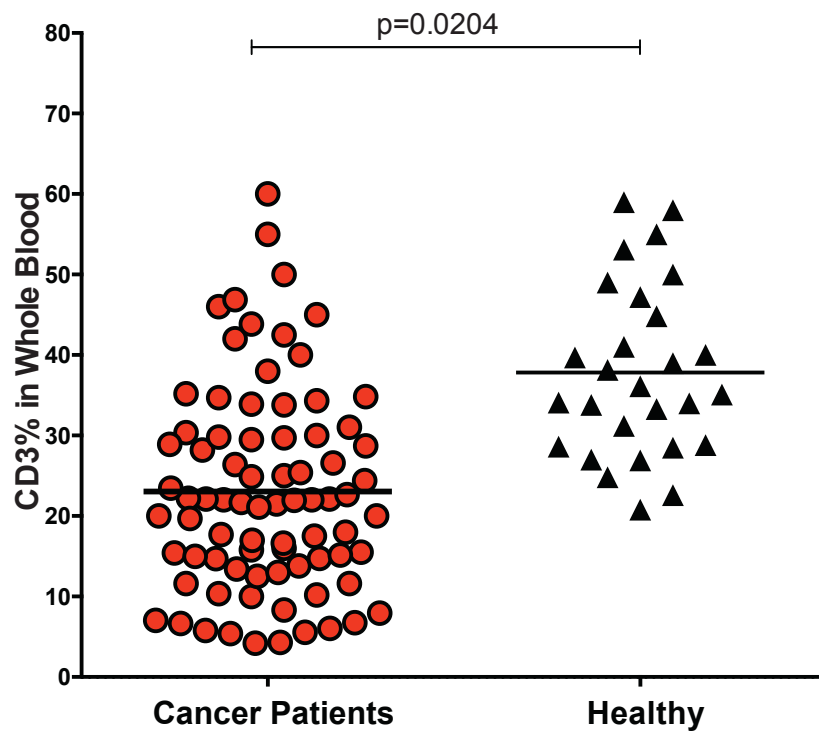**B**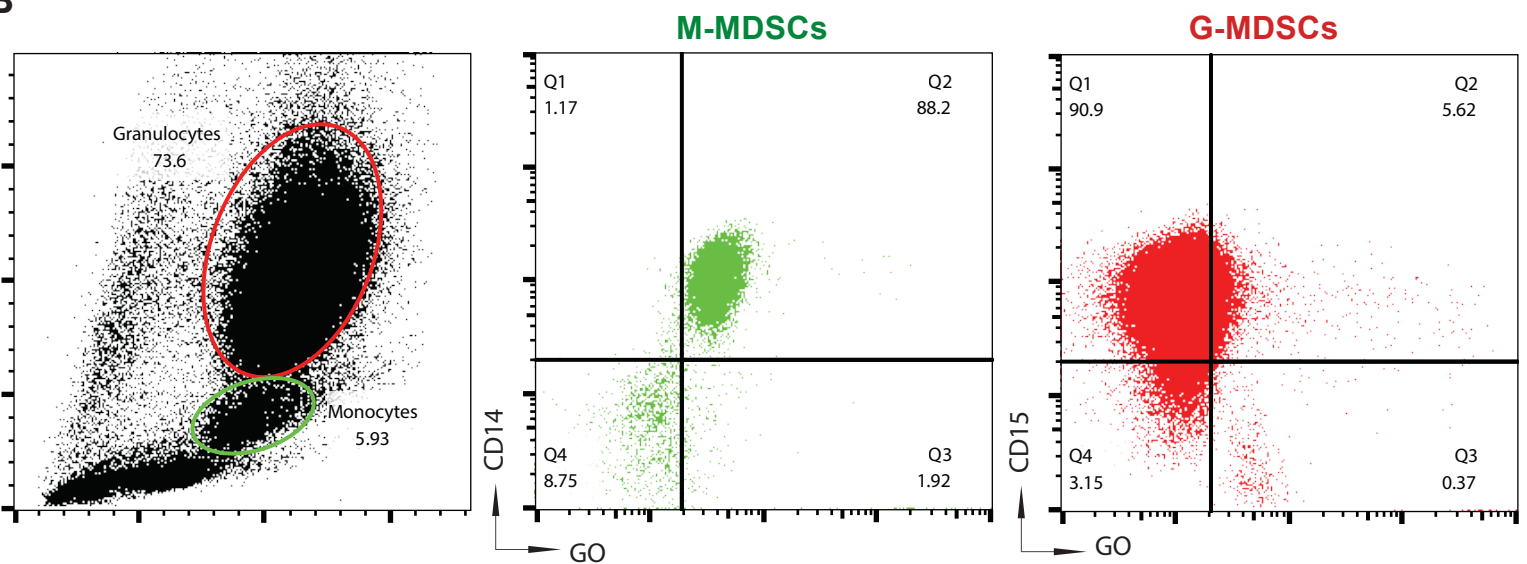**C**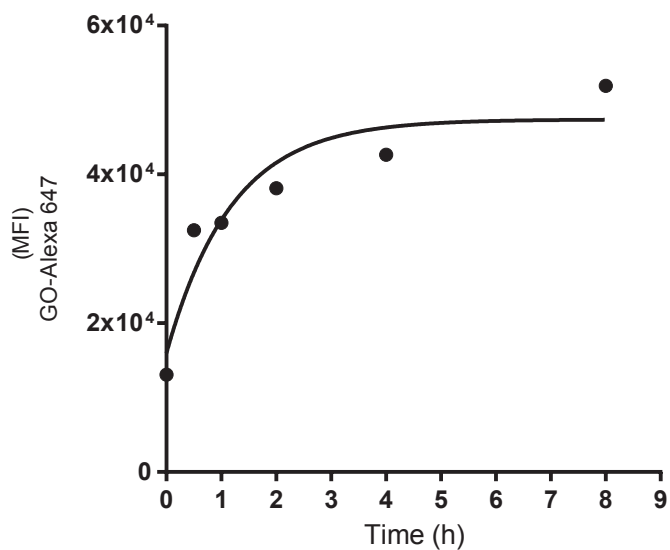**D**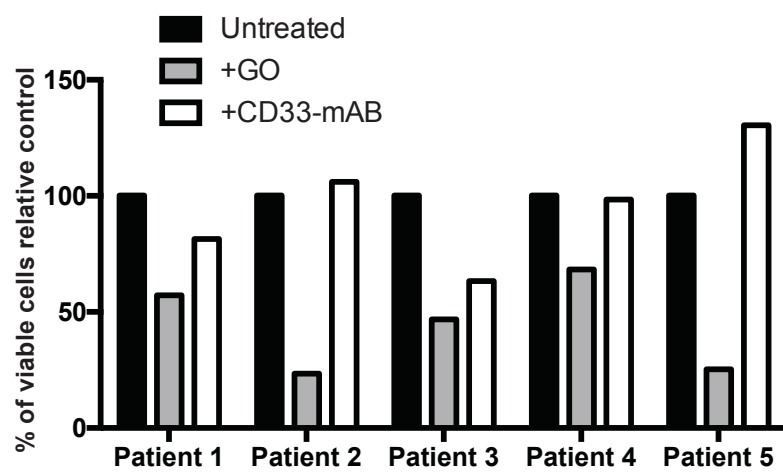

Supplement: Supplementary Fig. 3 — Myeloid and T cell populations in the blood of patients. A) Percentage of CD3+ T cells in the blood of cancer patients at diagnosis (n = 51) assessed by flow cytometry B) Flow cytometry gating of patient's whole blood demonstrating GO-ALEXA-647 staining of MDSCs. Representative of 5 individual experiments C) Gemtuzumab ozogamicin labelled with ALEXA-647 is internalised into MDSCs. Flow cytometric representation of internalised fluorescence over time. Representative of 3 independent experiments. D) The cytotoxicity of unconjugated gemtuzumab antibody (2 μg/ml) against CD33+ patient-derived MDSCs from different cancer subtypes, compared to gemtuzumab ozogamicin (2 μg/ml) and untreated controls, as assessed by flow cytometry with propidium iodide staining. [file mmc3.pdf]

**A**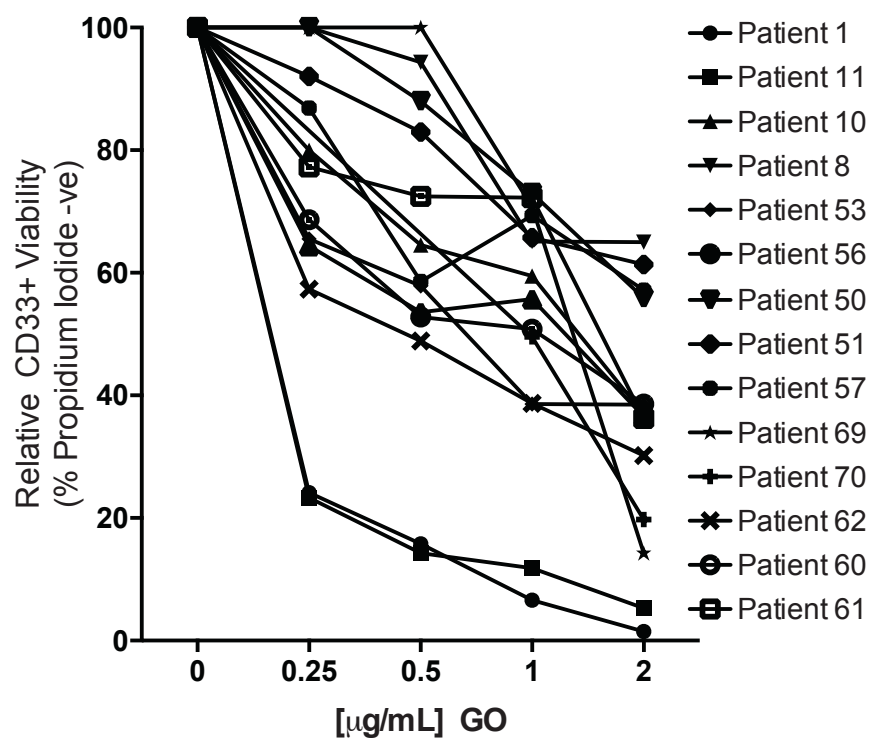**B**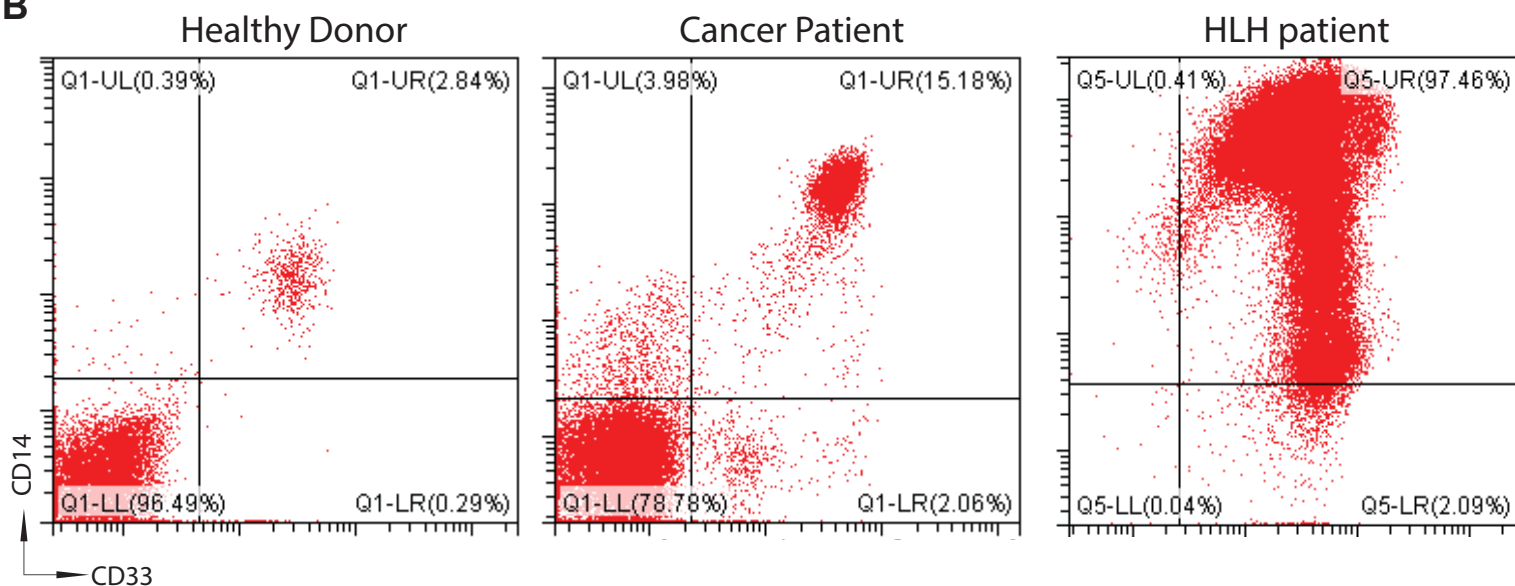**C**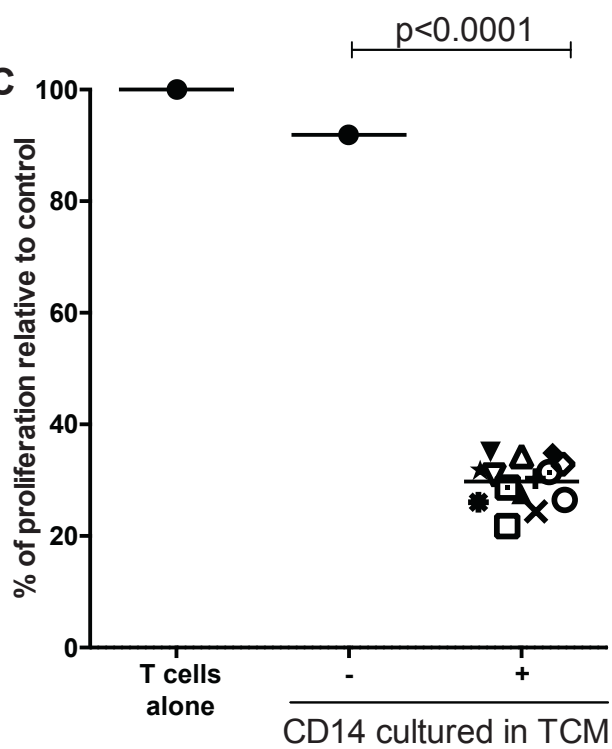**D**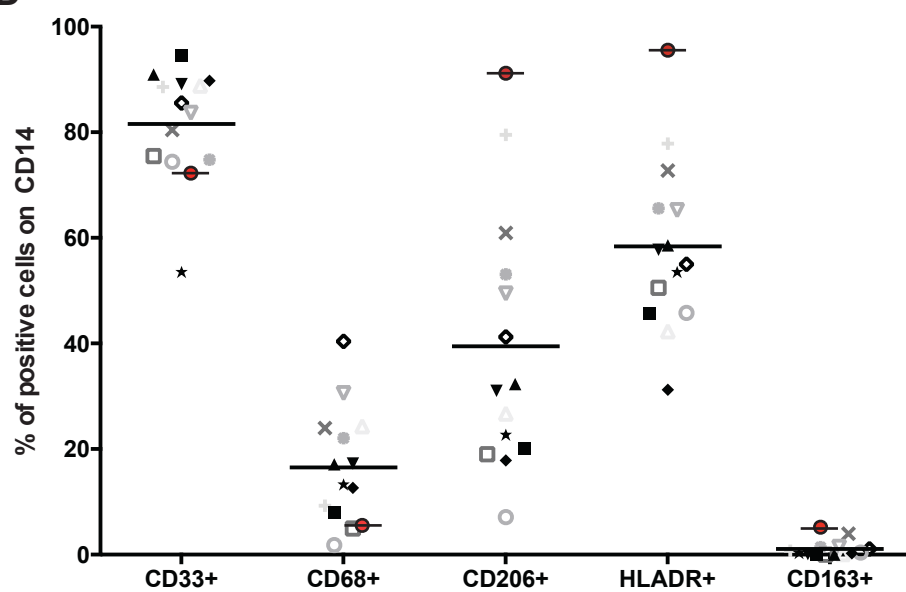

Supplement: Supplementary Fig. 4 — Gemtuzumab ozogamicin has activity against MDSCs. A) Dose-responses curves of Gemtuzumab ozogamicin cytotoxicity against CD33+ patient-derived MDSCs from different cancer subtypes, compared to untreated, as assessed by flow cytometry with propidium iodide staining B) Immunophenotyping of cancer patient and HLH patient blood demonstrating expansions of CD33 + CD14 M-MDSCs, by flow cytometry. Representative of n = 124 patients and 41 healthy donors C) T cell proliferation is suppressed following culture with CD33 + CD14+ tumour-polarised MDSCs. T cells and CD33 + CD14+ tumour-polarised MDSCs were co-cultured at a ratio of 1:0.5 and compared to CD33 + CD14+ monocytes. Mean of T cells and unpolarised MDSC proliferation shown as controls. D) The percentage of CD68+ CD14+ cells is increased following tumour polarisation, compared to the mean of unpolarised cells (RED). HLA-DR is also downregulated compared to unpolarised cells, as assessed by flow cytometry. [file mmc4.pdf]

**A**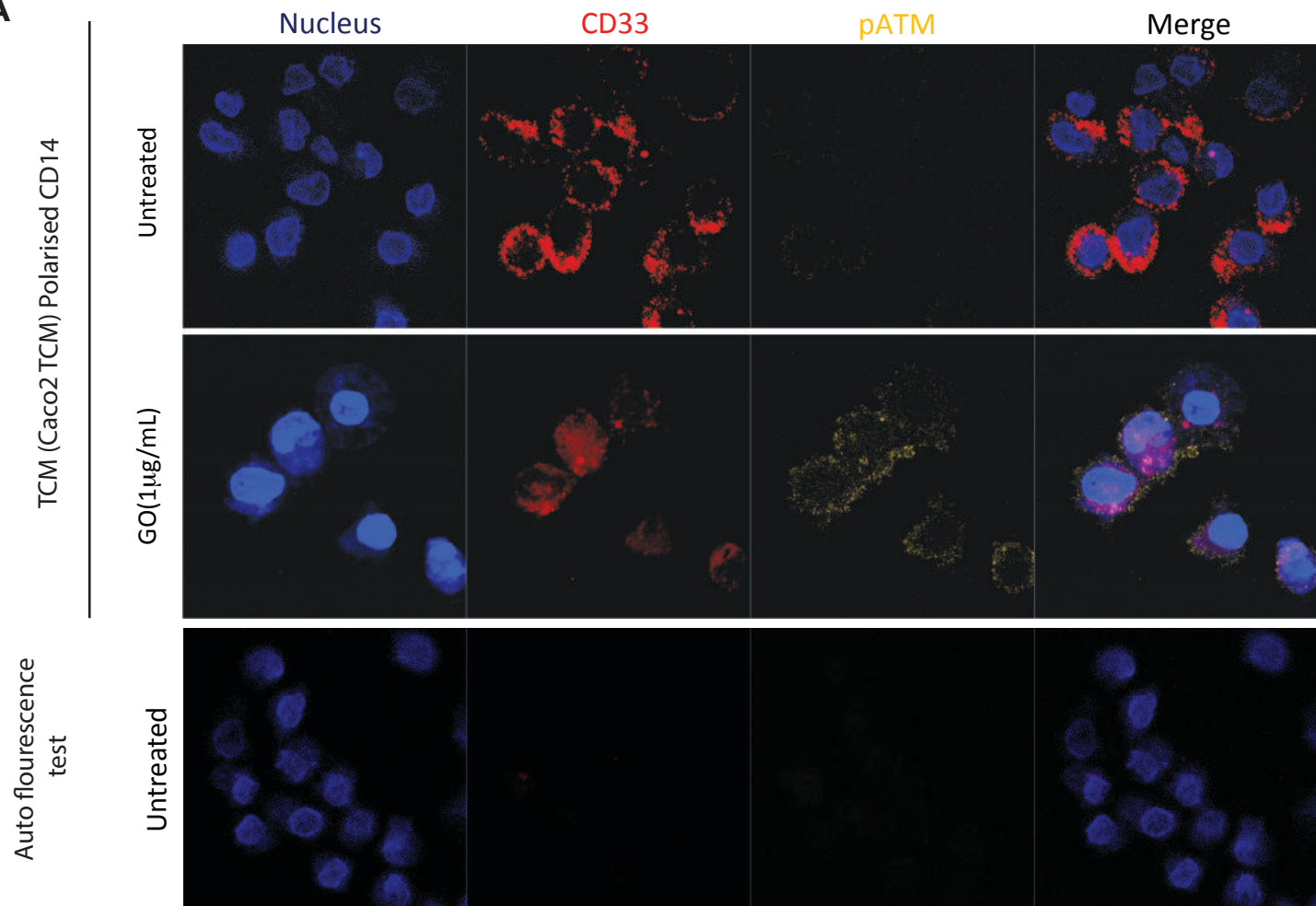**B**

M-MDSCs Untreated

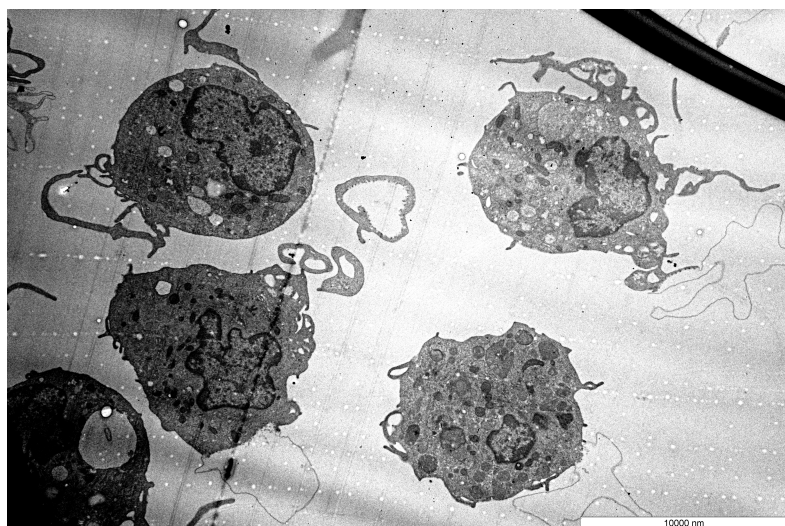M-MDSCs GO-Treated (1 $\mu$ g/ml)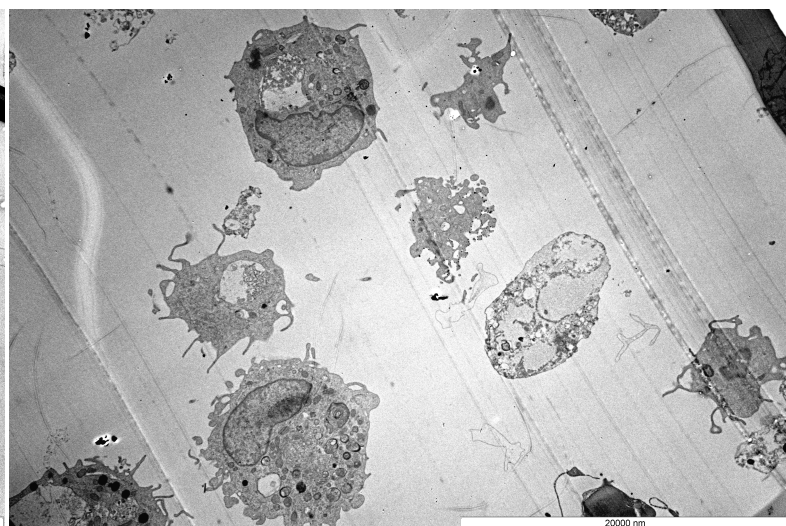

Supplement: Supplementary Fig. 5 — Gemtuzumab ozogamicin leads to MDSC cell death. A) Immunofluorescence staining of GO-treated (1 μg/ml) CD33+ MDSCs from the blood of patients showing increased p-ATM B) Transmission electron microscopy shows loss of cell membrane integrity, nuclear condensation, and blebbing consistent with apoptotic cell death after 1 μg/ml GO treatment. [file mmc5.pdf]

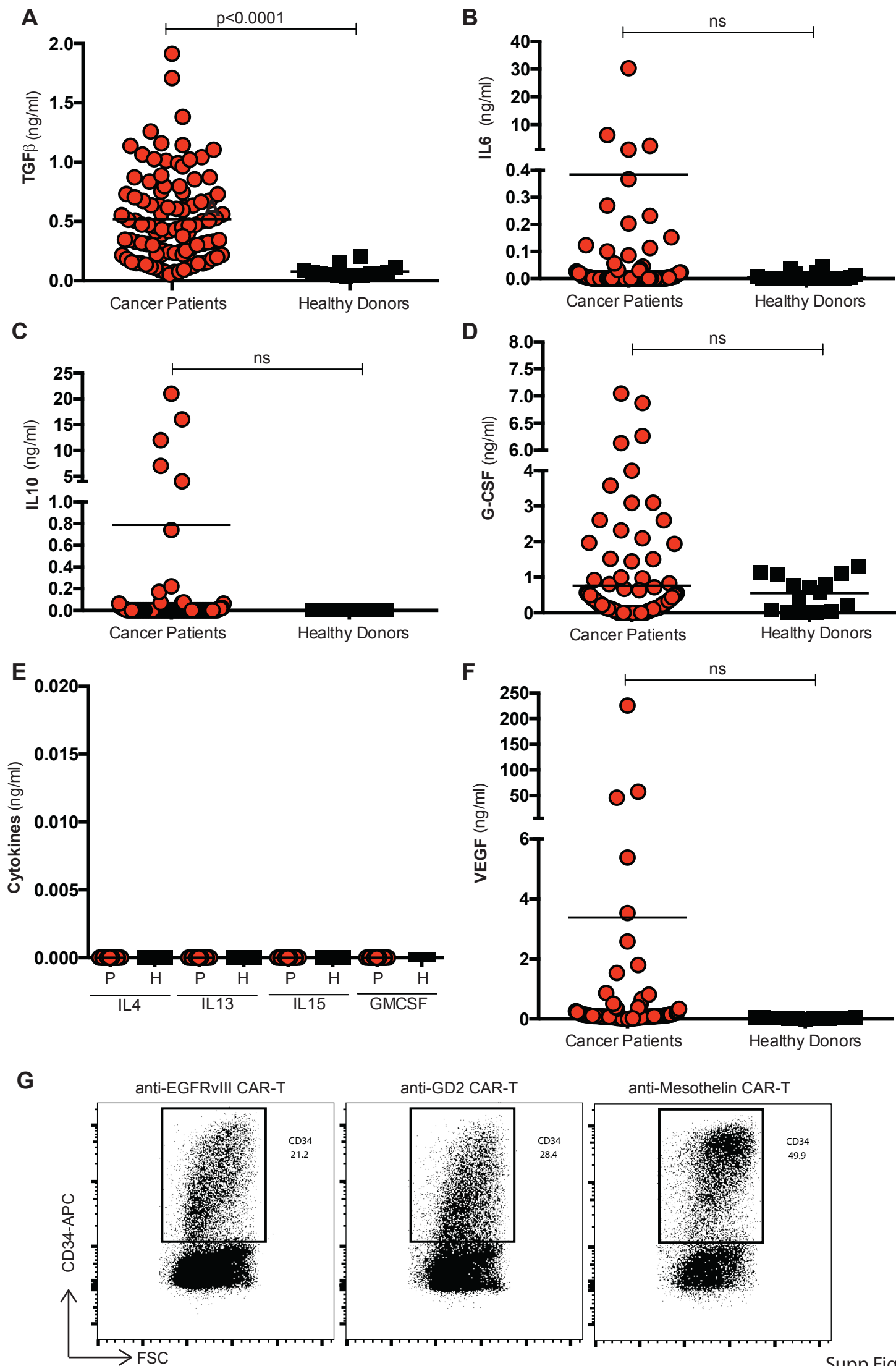

Supplement: Supplementary Fig. 6 — Systemic immunosuppressive cytokine environment in cancer patients. A-F) ELISAs detecting cytokine concentrations in the blood of cancer patients at diagnosis (n = 50), compared to healthy controls G) Flow cytometry demonstrating efficiency of CAR-T transduction of T cells prior to enrichment, gating on tCD34. [file mmc6.pdf]

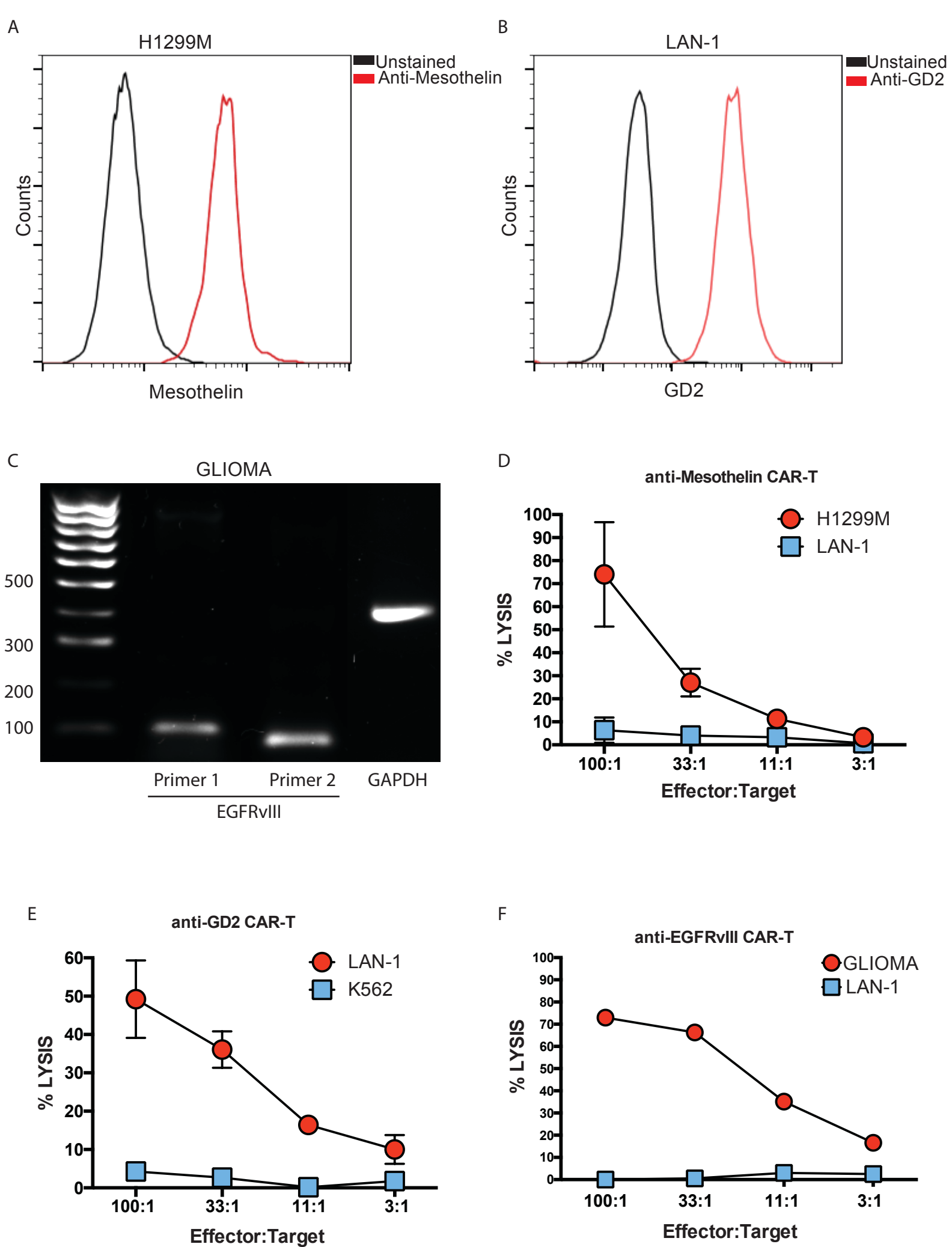

Supplement: Supplementary Fig. 7 — CAR-T cell cytotoxicity against antigen expressing tumour cell targets. A) Expression of mesothelin by Meso-ACC cell line, as determined by flow cytometry B) Expression of GD2 by LAN-1 cell line, as determined by flow cytometry C) Expression of EGFRvIII by glioma tumour cells from a patient, as determined by RT-PCR. Chromium (51Cr) release assay demonstrating antigen- specific killing of tumour cell targets (RED) by CAR-T cells. Minimal killing is seen against tumour cells which don't express the corresponding antigen (BLUE). H1299M (Mesothelin positive), LAN-1 (GD2 positive, mesothelin and EGFRvIII negative), K562 (GD2 negative), Glioma (EGFRvIII positive). [file mmc7.pdf]

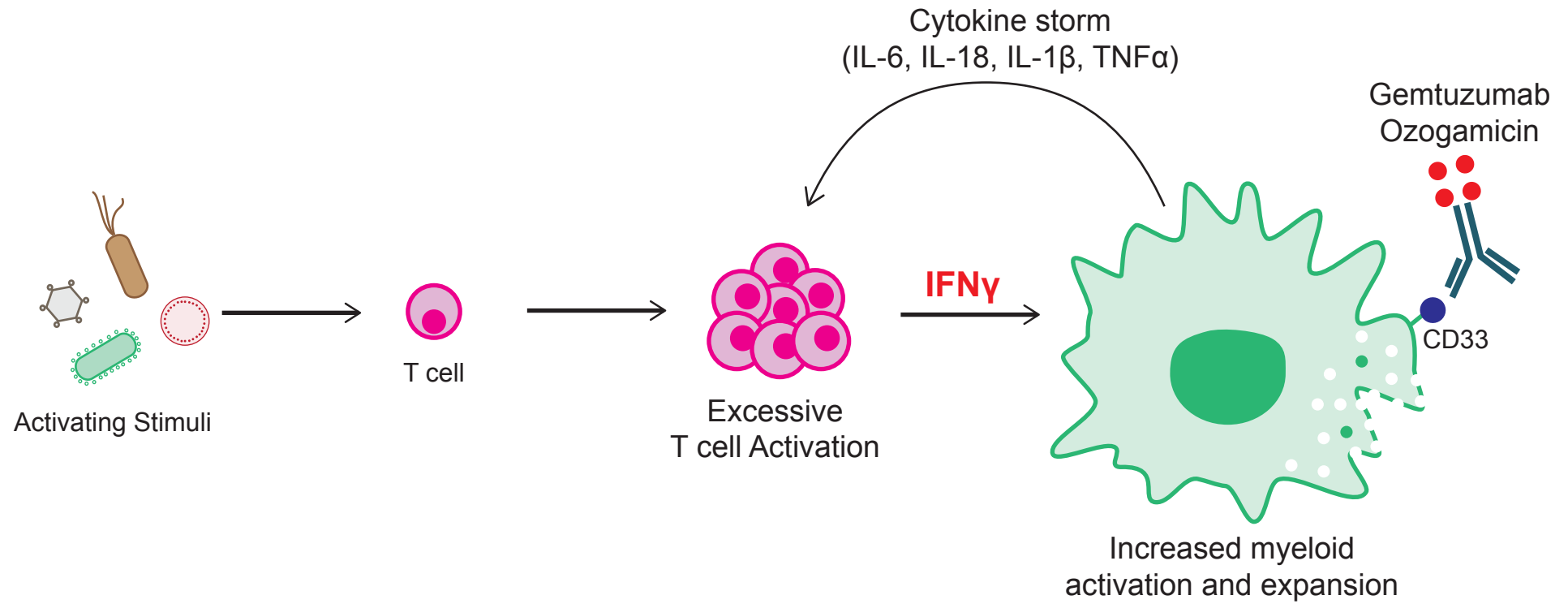

Supplement: Supplementary Fig. 8 — Schematic illustrating the potential of Gemtuzumab ozogamicin to target myeloid cells in HLH/MAS which release pro-inflammatory cytokines and drive pathological T cell responses. [file mmc8.pdf]
