# Supplementary material for: MDSC targeting with Gemtuzumab ozogamicin restores T cell immunity and immunotherapy against cancers
Source: eBioMedicine. 2019 Aug 25;47:235–46. doi: 10.1016/j.ebiom.2019.08.025 (PMC6796554; doi:10.1016/j.ebiom.2019.08.025)
Supplement: Supplementary Fig. 2 — Bone marrow histology of patients with secondary Haemophagocytic Lymphohistiocytosis. Immunohistochemical staining with Haematoxylin and Eosin in the bone marrow of patients (n = 8) with secondary Haemophagocytic Lymphohistiocytosis. [file mmc2.pdf]

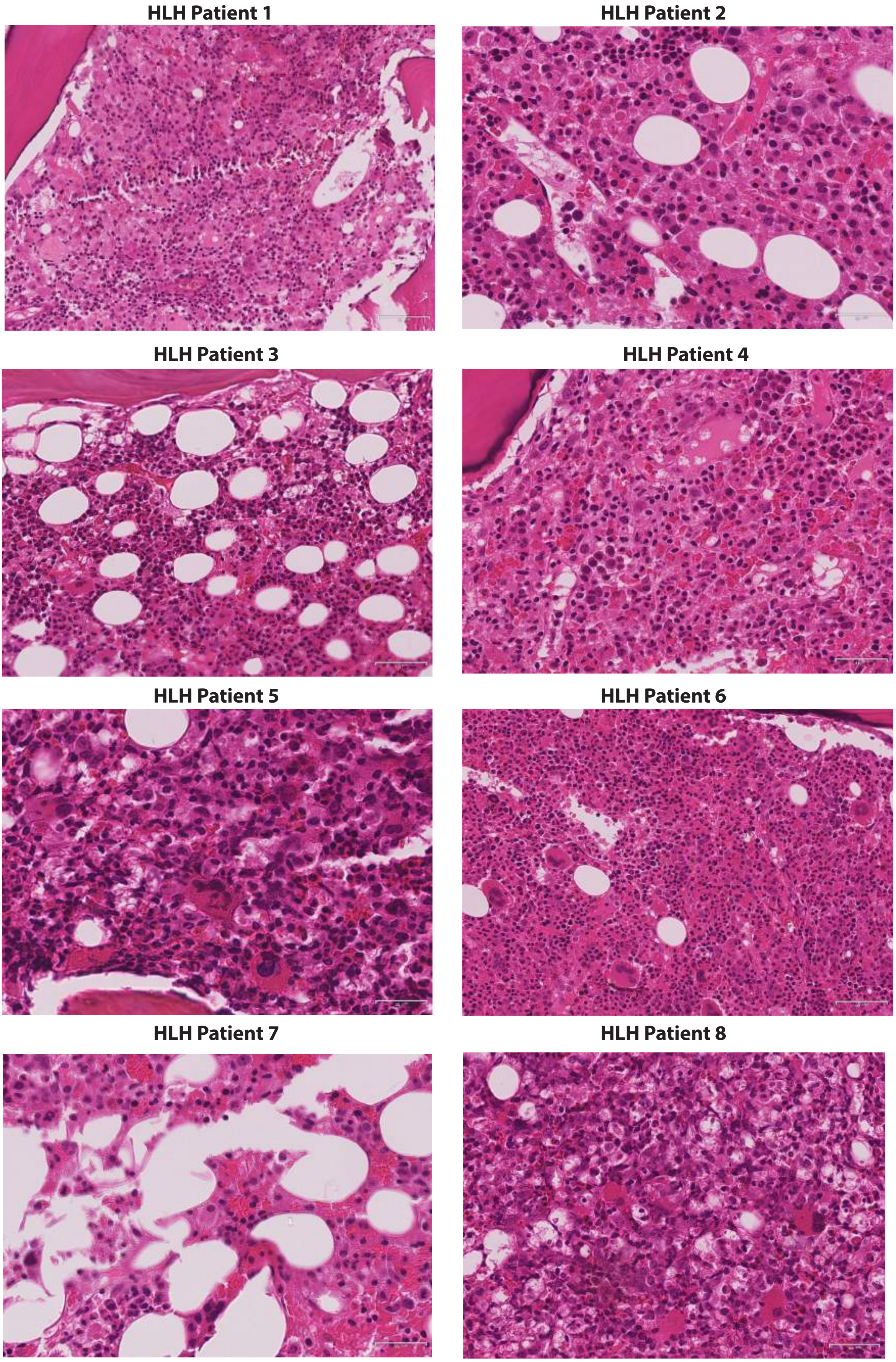

Supp Figure 2
